# Economic Evaluations of Gestational Diabetes Mellitus Screening: A Systematic Review

**DOI:** 10.2188/jea.JE20190338

**Published:** 2021-03-05

**Authors:** Xiuting Mo, Ruoyan Gai Tobe, Yoshimitsu Takahashi, Naoko Arata, Tippawan Liabsuetrakul, Takeo Nakayama, Rintaro Mori

**Affiliations:** 1 Department of Health Policy, National Center for Child Health and Development, Tokyo, Japan; 2 Department of Health Informatics, Kyoto University School of Public Health, Kyoto, Japan; 3 Department of Empirical Social Security Research, National Institute of Population and Social Security Research, Tokyo, Japan; 4 Division of Women’s Health and Reproductive Endocrinology, National Center for Child Health and Development, Tokyo, Japan; 5 Epidemiology Unit, Prince of Songkla University, Songkla, Thailand

**Keywords:** gestational diabetes mellitus screening, economic valuation, review

## Abstract

**Background:**

This study aims to find evidence of the cost-effectiveness of gestational diabetes mellitus (GDM) screening and assess the quality of current economic evaluations, which have shown different conclusions with a variation in screening methods, data sources, outcome indicators, and implementation in diverse organizational contexts.

**Methods:**

Embase, Medline, Web of Science, Health Technology Assessment, database, and National Health Service Economic Evaluation Database databases were searched through June 2019. Studies on economic evaluation reporting both cost and health outcomes of GDM screening programs in English language were selected, and the quality of the studies was assessed using Drummond’s checklist. The general characteristics, main assumptions, and results of the economic evaluations were summarized.

**Results:**

Our search yielded 10 eligible economic evaluations with different screening strategies compared in different settings and perspectives. The selected papers scored 81% (68–97%) on the items in Drummond’s checklist on average. In general, a screening program is cost-effective or even dominant over no screening. The one-step screening, with more cases detected, is more likely to be cost-effective than the two-step screening. Universal screening is more likely to be cost-effective than screening targeting the high-risk population. Parameters affecting cost-effectiveness include: diagnosis criteria, epidemiological characteristics of the population, efficacy of screening and treatment, and costs.

**Conclusions:**

Most studies found GDM screening to be cost-effective, though uncertainties remain due to many factors. The quality assessment identified weaknesses in the economic evaluations in terms of integrating existing data, measuring costs and consequences, analyzing perspectives, and adjusting for uncertainties.

## INTRODUCTION

Gestational diabetes mellitus (GDM) is defined as any degree of glucose intolerance with onset or first recognition during pregnancy. Approximately 17.8% (range, 9.3–25.5%) of pregnant women suffer complications due to GDM, depending on the epidemiological characteristics of the population investigated and diagnostic tests employed.^1^ GDM has become an important public health issue and is responsible for increased risks of maternal, prenatal, and neonatal complications, such as type 2 diabetes mellitus (T2DM) and cardiovascular disease in mothers and obesity and long-term metabolic syndrome in their offspring,^2^ potentially increasing the economic burden of healthcare. It is possible to manage GDM during pregnancy using nutritional management, insulin treatment, or oral hypoglycemic agent, with the primary goal of maintaining blood glucose within normal levels. Moreover, monitoring and prevention of T2DM in women with prior GDM in the postnatal period is also important in reducing the long-term disease burden. Women with GDM were found to have a higher risk of developing postpartum diabetes.^3^ For the offspring, diabetes, cardiovascular alterations, and/or obesity in adulthood are the lifelong consequences of intrauterine exposure to increased glucose.^4^^,^^5^

There are many studies on the economic evaluations of GDM management during both prenatal and postnatal periods.^6^^,^^7^ To manage GDM, many countries have implemented a screening program to identify asymptomatic pregnant women. However, the definition of GDM, the target population, and clinical practices vary among studies.^8^ GDM screening protocols are of two types and their modifications: a two-step method (a first-step glucose challenge test [GCT] and a second-step oral glucose tolerance test [OGTT]) that diagnoses based on two or more abnormal values (5.3 mmol/L while fasting, 10.0 mmol/L 1 hour postprandial, and 8.6 mmol/L 2 hours postprandial) on OGTT and a one-step method that recommends a 75 g OGTT test without a 50 g GCT before and has a simpler, one-abnormal-value diagnosis criteria.^9^ From an economic evaluation perspective, different conclusions have been drawn due to the different screening methods; data sources, outcomes, and interventions vary widely across studies examining disparate systems in diverse organizational contexts. Therefore, this study aims to systematically review the evidence on the cost-effectiveness of GDM screening and perform a quality assessment.

## METHODS

### Literature search

We conducted two independent searches of the related literature through June 2019 by Mo and Gai. We searched Embase, Medline, Web of Science, Health Technology Assessment (HTA) database, and National Health Service Economic Evaluation Database (NHSEED) for studies related to “economic evaluation of gestational diabetes screening” using the following search strings in Embase, MEDLINE, Web of Science, and NHSEED: TS=(((diabet* AND (pregnanc* OR pregnant OR gestation* OR wom?n OR female* OR mother*)) OR gdm) AND (screening* OR diagnos* OR glucose tolerance*) AND ((cost* AND (effectiveness OR benefit* OR utility)) OR (economic AND evaluation*))). In HTA, the search strings used were: (((diabet* and (pregnanc* or pregnant or gestation* or wom?n or female* or mother*)) or gdm) and (screening* or diagnos* or glucose tolerance*)). We did not select a time range for the search. All citations were imported into EndNote for further screening.

### Screening of studies

The screening was conducted by Mo under the supervision of Gai. The studies were screened in three steps. First, all duplicate papers were found using EndNote; second, all the apparently relevant studies were selected by reviewing their titles and abstracts; and last, the full texts were read. The inclusion criteria were: 1) cost-effectiveness analysis, reporting both input of health resources and output of health gains; 2) studies of screening programs for detecting GDM during pregnancy among women of reproductive age; and 3) original studies involving decision modelling or other mathematical methodologies to deal with uncertainties in cost-effectiveness. The studies that only reported cost or effectiveness and did not discuss the trade-off on marginal costs or health gains were excluded (see PRISMA 2009 Checklist in eTable 1).

### Quality assessment and critical appraisal

We assessed the quality of the included studies using the Assessing Economic Evaluations Checklist from the *Methods for the Economic Evaluation of Health Care Programmes*,^10^ which contains 10 major questions on the following: answerable question posed; competing alternatives given; effectiveness of the programs or services established; costs and consequences identified; costs and consequences measured accurately, credibly, and adjusted for differential timing; incremental analysis performed; uncertainty characterized; and discussions including all issues of concern to the users. Each question contains several sub-questions. The responses available are: “Yes,” “Partially yes,” “No,” and “Can’t tell.” A “Yes” is equivalent to a full score, a “No” has a value of 0, and a “Partially yes” or “Can’t tell” has a value of half a point each. For each “Not Applicable” (N.A.) response, the corresponding sub-question is disregarded (eTable 2). The quality of one paper^11^ was independently assessed thrice and the divergences and cases of “Partially yes” and “Can’t tell” were fully discussed by Mo, Agari Takahiro, and Naito Yumi. Then, the rest of the evaluation was completed by Mo.

## RESULTS

### Study selection

**Figure 1.**
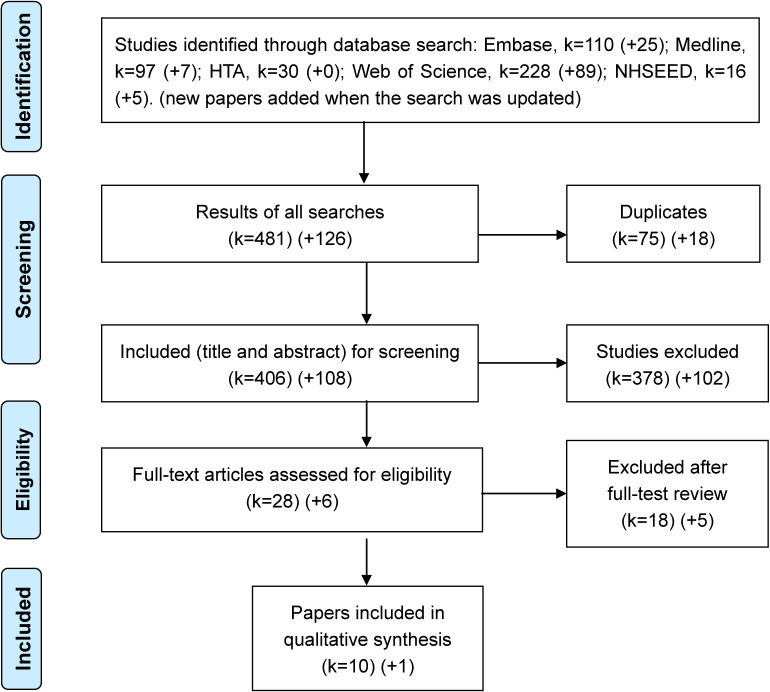
Items selected for systematic review and meta-analyses (PRISMA). Embase, Excerpta Medica Database; HTA, Health Technology Assessment; Medline, Medical Literature Analysis and Retrieval System Online; NHSEED, National Health Service Economic Evaluation Database. The search was updated in June 2019.

Embase, MEDLINE, HTA, Web of Science, and NHSEED yielded a total of 136, 104, 30, 317, and 21 articles, respectively. The search results were updated in June 2019. In all, 608 studies were identified. We excluded 93 duplicated studies and 480 articles that did not discuss GDM screening during pregnancy or only covered cost estimates or effectiveness. Fourteen poster or abstract sessions^12^^–^^25^ and 10 other types of articles^26^^–^^35^ were excluded (see Table 1). Of the remaining 11 articles, two^36^^,^^37^ that reported similar results using the same model were considered a single study. Finally, 10 studies^11^^,^^36^^–^^45^ were included and analyzed (Figure 1).

**Table 1.** Characteristics of excluded studies at second-level screening

First author	Year	Reason for exclusion
Cade TJ^26^	2019	Cost and effectiveness were separately discussed
Liang SQ^12^	2019	Only abstract available
Duarte A^13^	2018	Only abstract available
Li LJ^14^	2018	Only abstract available
Sortso C^27^	2018	Only related to cost calculation
Rodrigues^28^	2017	Only compared cost per case detected, did not include trade-off in marginal cost or health gain
Walker AR^15^	2017	Only abstract available
Pearson LJ^16^	2016	Only abstract available
Ming WK^17^	2016	Only abstract available
Zhang L^18^	2015	Only abstract available
Quitian H^19^	2015	Only abstract available
Duran A^29^	2014	Only compared cost saving per case, did not include trade-off in marginal cost or health gain
Chen PY^20^	2014	Only abstract available
Gillespie^30^	2012	Only related to cost per case detected
Werner EF^21^	2012	Only abstract available
Reel M^22^	2011	Only abstract available
Van Leeuwen M^23^	2009	Only abstract available
Lee S^24^	2008	Only abstract available
Thung S^25^	2007	Only abstract available
Ayach W^31^	2006	Cost and effectiveness were separately discussed
Rey E^32^	2004	Cost and effectiveness were separately discussed
Larijani B^33^	2004	Only related to cost per case screened/detected
Di Cianni G^34^	2002	Only related to cost per case detected
Weiner CP^35^	1986	Only related to cost per case identified

### General characteristics of the economic evaluations

Four of the included studies^39^^,^^41^^,^^42^^,^^45^ used TreeAge and three used Microsoft Excel^37^^,^^40^^,^^43^ to construct a decision tree for their economic model. Their general characteristics are summarized in Table 2. The first study was published in 2002 by Poncet^45^ and the next one in 2005.^44^ The remaining eight studies were published between 2011 and 2017.^11^^,^^36^^–^^43^ Four evaluations were from the United States,^36^^,^^41^^,^^42^^,^^44^ three were from Europe (United Kingdom,^38^ Ireland,^11^ and France^45^), one was from New Zealand,^40^ and the remaining two were from Asia (Singapore,^39^ India, and Israel^36^^,^^37^). Most of the studies used cost-utility analysis (CUA), where utility is measured in quality-adjusted life years (QALYs) or disability-adjusted life years (DALYs). Two studies used cost-effectiveness analysis (CEA)—one considered cases detected as the outcome,^40^ while the other used prevented pregnancy complications like, macrosomia, prematurity, perinatal mortality, and hypertensive disorders as the outcome.^45^ In terms of economic evaluation, five of the publications were from a healthcare perspective (third-party payer),^11^^,^^38^^,^^40^^,^^41^^,^^43^ one was from the payers’ perspective,^39^ and two were from a societal perspective.^42^^,^^44^ The remaining three studies did not clarify their perspective.^36^^,^^37^^,^^45^ One study was supported by a pharmaceutical company (Novo Nordisk),^36^^,^^37^ one failed to mention any funding,^42^ and the others were supported by public funding.

**Table 2.** Study overview of published economic evaluations of GDM screening

First authors, year	Country/ population	Journal	Type of economic evaluation	Perspective	Funding	Comparators (criteria/cutoff value)
Jacklin PB, 2017	UK	BMJ	CUA (QALY)	Healthcare	the National Institute for Health and Care Excellence	1. no screening; 2. NICE 2015 diagnostic threshold 3. IADPSG threshold. (universal screening on baseline; population with or without risk factors for subgroup analysis)
Danyliv A, 2016	Ireland	Diabetologia	CUA (QALY)	Healthcare	the Health Research Board of Ireland	1. no screening; 2. universal 2 h 75 g OGTT (IADPSG) at a GP practice; 3. universal 2 h 75 g OGTT (IADPSG) at hospital-based screening
Chen PY, 2016	Singapore	Asia Pac J Public Health	CUA (QALY)	Payers	Health Services and Systems Research Program at Duke-NUS Graduate Medical School and KK women’s and Children’s hospital from Singhealth Group	1. no screening; 2. universal 2 h 75 g OGTT (IADPSG); 3. targeted 2 h 75 g OGTT (IADPSG) based on risk factors following NICE guidelines
Coop C, 2015	New Zealand	BMJ	CEA	Healthcare	the Ministry of Health to support the development of a clinical practice guideline	1. at first booking HbA1c test + at 24–28 weeks (IADPSG) 2 h 75 g OGTT; 2. at first booking HbA1c test + at 24–28 weeks 1 h 50 g GCT ± 2 h 75 g OGTT (IADPSG)
Werner EF, 2012	US	Diabetes Care	CUA (QALY)	Healthcare	Department of Obstetrics, Gynecology, and Reproductive Sciences at the Yale School of Medicine	1. no screening; 2. at 24–28 weeks 1 h 50 g GCT ± 3 h 100 g OGTT (Carpenter and Coustan criteria); 3. at first booking FBG ± at 24–28 weeks 2 h 75 g OGTT (IADPSG)
Mission JF, 2012	US	Am J Obstet Gynecol	CUA (QALY)	societal	not mentioned	1. at 24–28 weeks 1 h 50 h GCT ± 3 h 100 g OGTT (Carpenter and Coustan criteria); 2. 2 h 75 g OGTT (IADPSG)
Lohse N, 2011 (Marseille E, 2013)	India and Israel	Int J Gynaecol Obstet (J Matern Fetal Neonatal Med)	CUA (DALY)	not mentioned	Novo Nordisk A/S	1. no screening; 2. 2 h 75 g OGTT and followed by prenatal intervention and postpartum preventive lifestyle prevention (IADPSG)
Round JA, 2011	UK	Diabetologia	CUA (QALY)	Healthcare	National Health Service in England and Wales	1. no screening; 2. 2 h 75 g OGTT; 3. FPG; 4. RBG; 5. GCT; 6. RBG ± 2 h 75 g OGTT; 7. FPG ± 2 h 75 g OGTT; 8. GCT ± 2 h 75 g OGTT. (NICE guidance, 2008)
Nicholson WK, 2005	US	Diabetes Care	CUA (QALY)	societal	Robert Wood Johnson Foundation and the National Institute of Diabetes and Digestive and Kidney Diseases	1. no screening; 2. 1 h 50 g GCT (140 gm/dl) ± 3 h 100 g GTT; 3. 2 h 75 g GTT (≥ 140 gm/dl); 4. 3 h 100 g GTT (95/180/155/145 mg/dl (0/1/2/3 h))
Poncet B, 2002	France	Eur J Obstet Gynecol Reprod Biol	CEA	not mentioned	Health ministry	1. no screening; 2. screening of high-risk women with 1 h 50 g GCT ± 3 h 100 g OGTT (Carpenter and Coustan criteria); 3. screening of all women with 1 h 50 h GCT ± 3 h 100 g OGTT (Carpenter and Coustan criteria); 4. screening of all women according to 2 h 75 g OGTT. (≥ 5.5/8 mmol/l (0/2 h))

Carpenter and Coustan criteria (1998), A 1 h glucose value ≥7.2 mmol/l indicates the need for a 100 g OGTT, and a diagnosis of GDM is made if in the fasting state: ≥5.3, 10.0, 8.6 or 7.8 mmol/L (0/1/2/3 h); CEA, cost effectiveness analysis; CUA, cost utility analysis; DALY, disability adjusted of life year; FPG, fasting plasma glucose; GCT, glucose challenge test; GDM, gestational diabetes mellitus; HbA1c test, haemoglobin A1c or glycated haemoglobin test; NICE, the National Institute for Health and Care Excellence; NICE 2015 threshold, GDM is defined as a FBG ≥5.6 mmol/L, 2 h 75 g OGTT ≥7.8 mmol/L; IADPSG, International Association of the Diabetes and Pregnancy Study Groups. FPG ≥5.1 mmol/L, a 75 g 1-hour OGTT ≥10.0 mmol/L, or a 75 g 2-hour OGTT ≥8.5 mmol/L; (O)GTT, (oral) glucose tolerance test; QALY, quality adjusted life year; RBG, random blood glucose.

The majority (8/10) of the selected studies included “no screening” for comparison.^11^^,^^36^^–^^39^^,^^41^^,^^44^^,^^45^ Large variations were found in the screening options, with three studies evaluating screening at different coverage rates (universal or high-risk targets),^38^^,^^39^^,^^45^ while one compared screening in different settings (GP practice or hospital-based).^11^ Two studies projected the long-term impact of screening on diabetes prevention.^36^^,^^37^^,^^41^ Most studies used the diagnostic criteria of the International Association of Diabetes and Pregnancy Study Groups (IADPSG) released in 2010^11^^,^^36^^,^^37^^,^^39^^–^^42^ or Carpenter and Coustan (CC)^41^^,^^45^; one used the 2008 guidelines of National Institute of Health and Clinical Excellence (NICE)^43^; and one compared different diagnostic thresholds (NICE guidelines of 2015 and IADPSG).^38^

### Main assumptions and results of economic evaluations

The major findings and sensitivity analysis results are summarized in Table 3, and the detailed input parameters of each study are presented in eTable 3 and eTable 4. As an important parameter, the GDM prevalence assumed in each study varied by area and criteria (0.016∼0.162). Most studies assumed universal screening uptake (100%) for comparison. Two studies considered the real uptake and acceptance rates,^11^^,^^40^ while three also considered the option of screening the high-risk population.^38^^,^^39^^,^^45^ Only one study (two articles) simulated long-term health effects on mothers and offspring.^36^^,^^37^ Most studies used the incremental cost-effectiveness ratio (ICER) to determine cost-effectiveness, with a diversified willingness to pay (WTP) threshold across study settings: £20,000 (suggested by NICE),^38^^,^^43^ €20,000/45,000 (Health Information and Quality Authority (HIQA) guidelines for the Republic of Ireland),^11^ $50,000,^39^^,^^44^ or $100,000^41^^,^^42^ (commonly referenced in American studies),^46^ per-capita GDP.^36^^,^^37^ (low resource countries usually refer to this threshold).^47^

**Table 3.** Main assumptions and results of published economic evaluations

**Author, year, ** **country**	Jacklin PB, 2017, UK	Danyliv A, 2016, Ireland	Chen PY et al, 2016, Singapore	Coop C, 2015, New Zealand	Marseille E, 2013, India and Isreal	Werner EF, 2012, US	Mission JF, 2012, US	Lohse N, 2011, India and Israel	Round JA, 2011, UK	Nicholson WK, 2005, US	Poncet B, 2002, France
**data sources/inputs**	3 datasets from HAPO, Norwich, and Atlantic Diabetes in Pregnancy	an RCT and literature, costs data were derived from HSE Payscales (2013)	literature, GUSTO birth cohort study and a hospital’s database	literature, National Women’s Annual Clinical Reports	literature and local data	literature and internal data	literature	literature, cost data were collected from pilot countries	literature	literature	literature, a prospective study and expert opinions
**GDM Prevalence**	NA	0.1236	0.0928	0.065	India: 0.091; Israel: 0.026	overt GDM: 0.016; 0.038 (CC); 0.162 (IADPSG)	GDM diagnosis with 1 h GCT: 0.054; missed GDM with 1 GCT: 0.124	0.1 in India and 0.026 in Israel	NA	0.04	0.03
**screening uptake**	100%	screening in GP: 52.69%; in hospital: 89.23%	high risk population (NICE guidelines: 38.97%) or 100%	initial HbA1c screening: 80%; GCT screening: 80%; 2 h OGTT screening following positive GCT: 90%; postnatal screening HbA1c: 80%	100% (intervention uptakes for GDM women is 80.0%, IGT women is 22.0%)	100%	100%	100% (intervention uptake is 50%)	100% (different rate assumed in scenario analysis)	100%	high risk population (rate unclear) or 100%
**Time horizon (life ** **expectancy for ** **mother and child ** **after birth, year ** **old)**	within 12 months	lifetime (51, 80)	lifetime (54, 82)	9-month time horizon	lifetime (39, 46 for India; 49, 57 for Israel)	lifetime (78 for healthy, 69 for untreated diabetes, 70 for treated diabetes)	lifetime (56.1, 77.2)	lifetime	lifetime (80)	lifetime	3-month
**Long-term health ** **effects on mothers/** **offspring included**	no/no	no/no	no/no	no/no	yes/yes (T2DM in mothers/offspring)	yes/no (postpartum screening and intervention to GDM women)	no/no	yes/yes (T2DM in mothers/offspring)	no/no	no/no	no/no
**Cost item included**	screening, GDM treatment, outcomes related costs	screening, GDM treatment, delivery, and NICU admissions	screening, GDM and preeclampsia treatment, delivery, and NICU admissions	screening, GDM and preeclampsia treatment, delivery, and NICU admissions	screening, GDM treatment and post-partum care costs	screening, GDM and preeclampsia treatment, delivery, newborn nursery care, care for permanent brachial plexus injury and intensive intervention to prevent diabetes	screening, GDM and preeclampsia treatment, delivery, newborn nursery care, care for permanent and transient BPI, hyperbilirubinemia, NICU admissions, and neonatal death	screening, GDM treatment and post- partum follow up and care costs	screening, treatment and professional costs	screening, maternal and infant care, lost productivity and wages	screening tests, obstetrical care, management of gestational diabetes mellitus, delivery care and sick leave, starting from the 24th week of gestation till discharge from maternity
**Cost evaluation**	£(UK, 2015)	€(Ireland, 2013)	$(USD, 2015)	$(NZ, 2013)	$(international dollar, 2011)	$(USD, 2011)	$(USD, 2012)	$(USD, 2010)	£(UK, 2009)	$(USD, 2003)	€(unclear)
**Health outcomes ** **evaluation**	ICER, £/QALY gain	ICER, €/QALY gain (mother and child post-delivery until death)	ICER, $/QALY gain (mother and child post-delivery until death)	(deemed as) ICER, $/case detected	ICER, $/DALY averted (mother and child post-delivery until death)	ICER, $/QALY gain (mother and child post-delivery until death)	ICER, $/QALY gain (mother and child post-delivery until death)	ICER, $/DALYs averted (mother and child post-delivery until death)	ICER, £/QALY loss due to a serious perinatal complication (mother and child post-delivery until death)	ICER, $/QALY gain (mother and child post-delivery until death)	(I)CER, €/case prevented; (incidence of macrosomia, prematurity, perinatal mortality, hypertensive disorders)
**CE Thresholds**	£20,000/QALY (suggested by NICE)	two ceiling ratios recommended levels: €20,000 and €45,000/ QALY (HIQA recommendation)	$50,000/QALY (Weintraub, 2008)	not mentioned	per-capita GDP 2010 of India: $3,500, and Israel: $29,800	$100,000/QALY (Shiroiwa, 2010)	$100,000/QALY (Caughey, 2005)	per-capita GDP 2010 of India: $3,400, and Israel: $29,500	£20,000/QALY (suggested by NICE)	$50,000/QALY (Russell, 1996)	NA
**Discounting rate**	3.5% for eff	5% for eff	3% for eff and cost	N.A.	3% for cost	3% for eff and cost	3% for eff	3% for cost	3% for eff	3% for eff and cost	NA
**Results**	NICE 2015 threshold vs no screening: £23,073/QALY; NICE 2015 vs WHO 2013: £37,669/QALY	GP/hospital screening vs no screening: dominated (NA); hospital vs GP: dominated (0.0006 QALY gained and €21.43 saved per case)	universal vs targeted: $10,630/ QALY gained; targeted vs no screening: $9,019/ QALY gained	2-step (HbA1c test + 2 h 75 g OGTT) vs 3-step (HbA1c test + 1 h 50 g GCT + 2 h 75 g OGTT): NZ$12,460 per case averted	screening vs no screening: India: $1,626 per DALY averted; Israel: $1,830 per DALY averted	when considered long term perinatal/ maternal benefits (or only considered perinatal outcomes): IADPSG strategy vs the current standard: $20,336 per QALY gained (not c-e); the current vs no screening: $16,689 per QALY gained ($543,119); the IADPSG vs no screening: $19,339 per QALY gained ($565,407).	the IADPSG (2 h 75 g OGTT) vs the routine strategy (1 h 50 h GCT + 3 h 100 g OGTT, CC criteria)): $61,503 per QALY gained	screening + postpartum intervention in India and Israel were DALYs averted (2.33; 3.10) and net savings ($78; $1,945)	When GDM risk is <1% then no screening/treatment strategy was cost- effective; where risk is between 1.0% and 4.2% FPG + OGTT was most likely to be cost-effective; and where risk is >4.2%, universal OGTT was most likely to be cost- effective.	compared with sequential strategy (50 g GCT + 100 g GTT), 100-g strategy was $32,372/QALY saved for maternal outcomes and $8,251/QALY saved for neonatal outcomes; the 75-g strategy and the no screening strategy were costlier and less effective	S2 (all with 50 g OGTT) vs S0 (no screening) costs 1.1 times more to obtain one additional effectiveness than S1 (high risk women with 50 g OGTT) vs S0; S3 (all with 75 g OGTT) vs S0 costs 3.7 times more expensive than S1 vs S0. (S2: high- risk by 50 g OGT had the most favorable CER)
**subgroup**	yes (women with or without risk factors; women from different datasets)	no	no	no	no	no	no	no (but results of India and Israel settings were displayed separately)	no (two sets of results were presented using data from two different studies)	no	no
**1-Way**	no	yes (varied ranges of discount rate, care cost ratio of two settings, and screening uptakes in two settings didn’t change the baseline results)	yes (any single variable associated with universal screening did not increase the ICER beyond the threshold)	no	yes (sensitive to the incidence of T2DM in GDM mothers and the cost and effectiveness of post-partum intervention)	yes (the model is robust and most sensitive to the probability that long- term intervention in iGDM patents would reduce progression to diabetes)	yes (sensitive to percentage of additional GDM diagnosis with 2 h OGTT, cost of 2 h OGTT and GDM treatment, efficacy of treatment)	no	no	yes (SA of parameters eg GDM prevalence, probabilities, utilities, costs were performed without unclear results description)	analysis type unknown; changing the values of the events and the values of the main outcome measures within the ranges did not change the results of the ICERs; when the cost is >5488.15 Euros, S4 is the most C-E
**2-way**	no	yes (changes of screening uptakes in both settings would change the baseline result)	yes (universal screening is preferred due to a high rate of GDM and/or screening and treatment are effective)	no	no (“multivariate SA” with unclear result description)	no	yes (cost ratio of 1 h GCT vs 2 h OGTT, efficacy of treatment vs cost of GDM treatment, probability of preeclampsia in group 2 with vs without treatment, probability of cesarean section in group 2 with vs without treatment)	no	no	no	
**PSA (Probabilistic ** **sensitivity analysis)**	yes	yes (cost ratio in two settings; did not change the results)	yes	no	no	yes (the IADPSG strategy was cost- effective in 96.4% of cases)	yes	no	yes (when GDM risk is <1%, no screening is preferred; when risk is between 1.0% and 4.2%, when FPG + OGTT is preferred; risk is >4.2%, OGTT is preferred)	no	
**EVPI (expected ** **value of perfect ** **information)**	no	no	no	no	no	no	no	no	no	no	
**CEAC/CEAF ** **(Cost-effectiveness ** **acceptability curve/** **frontier)**		yes (cost ratio: 0.9∼1,4 doesn’t change the results)	yes (only when WTP drops to $10,000/QALY, targeted screening is better than universal screening)	no	no	no	yes (a 75.5% probability that 2 h OGTT would be cost-effective)	no	no	no	no
**Scenario**	yes (WTP = £20,000/QALY)	yes (WTP = 20,000 or 45,000 per QALY)	no	yes (GDM prevalence and OGTT sensitivity and specificity)	yes (perinatal mortality for GDM- affected group, reduction in T2DM, receive post-partum rate)	no	no	yes (4 scenarios: 50% higher treatment cost, higher GDM prevalence, lower T2DM incidence in GDM, lower intervention efficacy, higher intervention costs, reduced T2DM is not permanent but delayed); in all scenarios, the intervention is either cost-saving or rather cost-effective)	yes (test acceptance rates vary instead of 100%: when GDM risk is <1.6%, no screening is the most C-E; when risk is between 1.60% and 3.6%, RBG + OGTT is preferred; when risk is >3.6%, GCT + OGTT is preferred)	no	no

CC criteria, Carpenter and Coustan criteria; DALY, disability of adjusted life year; FPG, fasting plasma glucose; GDP, gross domestic product; GUSTO birth cohort study, Growing up towards Healthy Outcomes birth cohort study; GDM, gestational diabetes mellitus; GCT, glucose challenge test; GDM, gestational diabetes mellitus; HAPO, Hyperglycaemia and Adverse Pregnancy Outcomes study; HbA1c test, haemoglobin A1c or glycated haemoglobin test; HIQA, Health Information and Quality Authority; IADPSG, International Association of the Diabetes and Pregnancy Study Groups; iGDM, infant of gestational diabetic mother; NICE, the National Institute for Health and Care Excellence; NICU, neonatal intensive care unit; (O)GTT, (oral) glucose tolerance test; QALY, quality adjusted life year; RBG, random blood glucose; RCT, randomized controlled trial; T2DB, type 2 diabetes mellitus; WTP, willingness to pay.

Compared with no screening, a screening strategy was considered dominated,^11^^,^^44^ cost-effective (C-E),^37^^,^^39^ or not C-E when the women were without risk factors (recommended by NICE; eg, polycystic ovary syndrome, previous stillbirth, or recurrent glycosuria) or when the GDM risk was less than 1%.^38^^,^^43^ The two-step approach described here was compared to the one-step approach (2 hr OGTT at 24–28 weeks), with the execution details differing slightly among the studies ((HbA1c test at first booking +) 1 hr GCT ± 2/3 hr OGTT). Compared with the two-step approach, the IADPSG (2010) diagnostic approach (one-step) cost more, detected more cases, and proved to be C-E (under baseline consumption)^40^^,^^42^ or C-E only when post-delivery care reduced diabetes incidence.^41^ Regarding the comparison of NICE (2015) and the IADPSG (2010) diagnostic thresholds, the lower FPG threshold of IADPSG detected more cases and was considered C-E only under a higher WTP (£30,000 per QALY).^38^

The coverage of the screening program tended to influence cost-effectiveness—universal screening or options with a higher screening uptake would be more C-E or even dominated compared with the alternative of only screening the high-risk population^41^ or a population with a low uptake.^11^ The GDM risk tended to affect cost-effectiveness, as well as we mentioned earlier, among women with or without lower risk factors (recommended by NICE), no screening strategy (or strict diagnostic threshold) was likely to be C-E.^38^^,^^43^

Regarding uncertainties, seven studies included a one-way sensitivity analysis (SA) and three reported a two-way SA. In all, five studies presented a probabilistic SA, among which, three presented results using cost-effectiveness acceptability curves/frontier. However, no study performed the expected value of perfect information analysis. Five studies conducted a scenario SA. Of all the existing SA parameters, the most influential ones include: the uptake of screening^11^^,^^43^; GDM prevalence^38^^,^^39^^,^^43^; effectiveness, sensitivity, and specificity of screening^39^^,^^42^; efficacy of treatment^39^^,^^42^; incidence of T2DM in GDM mothers^37^; cost and effectiveness of post-partum intervention^37^^,^^41^; cost of screening^42^; cost of GDM treatment^42^; and WTP^38^^,^^39^ in the respective studies.

### Quality assessment and critical appraisal

The quality scores for the 10 studies shown in Table 4 demonstrate that, on average, 81% (68–97%) of the items on Drummond’s checklist were addressed. Specific sub-question scores are shown in eTable 2. Most studies reported problems with Questions 3, 4, and 7. In Question 3, effectiveness based on previous randomized control trials (RCTs) and/or systematic overview required clarification. However, only two studies provided details of their search strategy and the rules for inclusion or exclusion.^41^^,^^45^ For Question 4, due to differences in the analytical perspective, the relevant costs and consequences were varied. Only one paper mentioned both capital and operating costs.^42^ Regarding Question 7, two papers did not consider long-term effectiveness,^40^^,^^45^ and five did not include discounting. Seven of the eleven papers scored over 80%.

**Table 4.** Critical assessment (Methods for the Economic Evaluation of Health Care Programmes: Assessing Economic Evaluations Checklist)

	Questions	Jacklin 2017	Danyliv 2016	Chen 2016	Coop 2015	Marseille 2013^a^	Werner 2012	Mission 2012	Lohse 2011^a^	Round 2011	Nicholson 2005	Poncet 2002	Average
1	Was a well-defined question posed in answerable form?	100%	100%	100%	100%	75%	100%	100%	75%	100%	100%	75%	93%
2	Was a comprehensive description of the competing alternatives given?	100%	100%	100%	100%	100%	100%	100%	100%	100%	100%	100%	100%
3	Was the effectiveness of the programmes or services established?	75%	83%	75%	75%	75%	100%	25%	75%	83%	50%	100%	74%
4	Were all the important and relevant costs and consequences for each alternative identified?	100%	83%	50%	33%	50%	33%	33%	67%	17%	83%	50%	50%
5	Were costs and consequences measured accurately in appropriate physical units prior to valuation?	100%	100%	100%	100%	100%	100%	100%	100%	100%	100%	100%	100%
6	Were costs and consequences valued credibly?	100%	100%	100%	100%	100%	100%	100%	75%	100%	75%	100%	95%
7	Were costs and consequences adjusted for differential timing?	N.A.	100%	50%	N.A.	50%	100%	50%	50%	100%	50%	N.A.	55%
8	Was an incremental analysis of costs and consequences of alternatives performed?	100%	100%	100%	0%	100%	100%	100%	100%	100%	100%	100%	90%
9	Was uncertainty in the estimates of costs and consequences adequately characterized?	100%	100%	83%	50%	83%	100%	83%	25%	100%	0%	17%	64%
10	Did the presentation and discussion of study results include all issues of concern to users?	100%	100%	100%	100%	83%	75%	67%	33%	83%	17%	83%	74%
	*Average*	96%	97%	86%	73%	82%	91%	76%	70%	88%	68%	81%	81%

^a^Lohse N, 2011 and Marseille E, 2013 were the same calculation.

## DISCUSSION

We reviewed the published economic evaluations of GDM screening and assessed quality in terms of options design, modelling, results, and parameters for sensitivity analysis for each paper, which were different from each other. Overall, screening is C-E or even dominant over no screening. Although the dominance of specific screening methods or targets could not be determined, recent studies have focused on screening using the 2 hr 75 g OGTT (IADPSG criteria) and compared it with no screening^11^^,^^36^^,^^37^^,^^39^ or with status quo (the two-step strategy).^40^^–^^42^ In the end, the method that results in more cases detected is likely to be C-E compared to the alternative on the conditions that postnatal care reduces diabetes incidence and that WTP increases. The results show that the one-step screening is comparatively more C-E than the two-step^41^^,^^42^ and the two-step is more C-E than the three-step.^40^ With a higher WTP, the option with a low diagnostic threshold (eg, the IADPSG criteria) is more C-E than its counterpart (eg, NICE 2015).^38^ A universal screening is C-E or dominant over no screening or a screening targeting the high-risk population (NICE),^11^^,^^41^ where a relatively large proportion of cases were detected. Conversely, the results of economic evaluation are different when targeting low-risk population.^38^^,^^43^ The dominance largely depends on the risks of the target individuals and the acceptability of the screening options.^38^^,^^43^

Other than the screening protocols and diagnosis criteria under different healthcare systems, and epidemiological characteristics of GDM (GDM prevalence and mortality) in the target population, other key factors that affect cost-effectiveness of the screening include: detection efficacy,^42^ long-term benefits attributable to early detection,^41^ treatment efficacy,^42^ and the cost of screening.^42^ In particular, the consideration of long-term outcomes has a significant influence on the results,^41^ which were not considered in almost all the studies examined, implying the importance of implementing effective postnatal interventions.

None of the studies compared different screening timings. Screening is usually performed at 24–28 weeks. Recent studies have suggested that GDM screening occur in the first trimester, accompanying other regular tests assessing a combination of maternal characteristics and biomarkers,^48^^–^^50^ since a previous study suggested that first-trimester HbA1c alone does not have sufficient sensitivity or specificity for diagnosis.^51^ Moreover, most studies were conducted in developed countries and evidence from low-income and middle-income countries is lacking.

Our review identified some methodological inconsistencies. For example, the difference between “ICER” and “CER,” definition of the C-E threshold, and discount rate were not clarified.^40^ Utilities and treatment effects were not clearly described either.^45^ While the type of SA is not considered in the quality assessment (Q9 in the uncertainty analysis), most studies conducted a deterministic and not a probabilistic SA, even though the latter can assess the cost-effectiveness of an target option at a certain threshold^52^ and characterize the combined effects of all parameter uncertainties simultaneously.^10^

Our review also identified a lack of clarity in the analytical perspective, types of study design, health gains, consideration of uncertainties, and discounting in some existing studies, which if included, would have made the results more reliable.^10^ Regarding reporting standards, the newly-launched guidelines for economic evaluation, such as the Consolidated Health Economic Evaluation Reporting Standards (CHEERS) statement,^53^ methodological guidelines proposed by NICE from the United Kingdom,^54^ and the International Society for Pharmacoeconomics and Outcomes Research,^55^ facilitate the creation of high-quality evidence.

### Conclusions

Our review shows that the screening program for GDM during pregnancy is C-E in general. The one-step screening, with more cases detected, is more likely to be C-E than the two-step screening. Universal screening is more likely to be C-E than screening targeting high-risk population. A higher screening uptake, more effective treatment, and postnatal interventions contribute toward improving cost-effectiveness. The quality assessment identified several weaknesses in performing and reporting economic evaluations and leaves us with lessons and research tasks for the future.

## APPENDIX A. SUPPLEMENTARY DATA

eTable 1. PRISMA 2009 Checklist for this study

eTable 2. Critical assessment including sub-questions

eTable 3. Treatment/intervention effects used in the baseline analyses of each study

eTable 4. Baseline utilities in each study
