# Supplementary material for: Economic Evaluations of Gestational Diabetes Mellitus Screening: A Systematic Review
Source: J Epidemiol. 2021 Mar 5;31(3):220–30. doi: 10.2188/jea.JE20190338 (PMC7878709; doi:10.2188/jea.JE20190338)
Supplement: Supplementary file 1 [file je-31-220-s001.pdf]

eTable 1. PRISMA 2009 Checklist for this study

Section/topic	#	Checklist item	Reported on page #
**TITLE**			
Title	1	Identify the report as a systematic review, meta-analysis, or both.	1
**ABSTRACT**			
Structured summary	2	Provide a structured summary including, as applicable: background; objectives; data sources; study eligibility criteria, participants, and interventions; study appraisal and synthesis methods; results; limitations; conclusions and implications of key findings; systematic review registration number.	2
**INTRODUCTION**			
Rationale	3	Describe the rationale for the review in the context of what is already known.	4-5
Objectives	4	Provide an explicit statement of questions being addressed with reference to participants, interventions, comparisons, outcomes, and study design (PICOS).	5
**METHODS**			
Protocol and registration	5	Indicate if a review protocol exists, if and where it can be accessed (e.g., Web address), and, if available, provide registration information including registration number.	NA
Eligibility criteria	6	Specify study characteristics (e.g., PICOS, length of follow-up) and report characteristics (e.g., years considered, language, publication status) used as criteria for eligibility, giving rationale.	5-6
Information sources	7	Describe all information sources (e.g., databases with dates of coverage, contact with study authors to identify additional studies) in the search and date last searched.	5
Search	8	Present full electronic search strategy for at least one database, including any limits used, such that it could be repeated.	5

Study selection	9	State the process for selecting studies (i.e., screening, eligibility, included in systematic review, and, if applicable, included in the meta-analysis).	5-6
Data collection process	10	Describe method of data extraction from reports (e.g., piloted forms, independently, in duplicate) and any processes for obtaining and confirming data from investigators.	5-6
Data items	11	List and define all variables for which data were sought (e.g., PICOS, funding sources) and any assumptions and simplifications made.	6
Risk of bias in individual studies	12	Describe methods used for assessing risk of bias of individual studies (including specification of whether this was done at the study or outcome level), and how this information is to be used in any data synthesis.	6
Summary measures	13	State the principal summary measures (e.g., risk ratio, difference in means).	6
Synthesis of results	14	Describe the methods of handling data and combining results of studies, if done, including measures of consistency (e.g., I^2) for each meta-analysis.	NA

Section/topic	#	Checklist item	Reported on page #
Risk of bias across studies	15	Specify any assessment of risk of bias that may affect the cumulative evidence (e.g., publication bias, selective reporting within studies).	7(Qualitative assessment and critical appraisal)
Additional analyses	16	Describe methods of additional analyses (e.g., sensitivity or subgroup analyses, meta-regression), if done, indicating which were pre-specified.	NA
**RESULTS**			

Study selection	17	Give numbers of studies screened, assessed for eligibility, and included in the review, with reasons for exclusions at each stage, ideally with a flow diagram.	7
Study characteristics	18	For each study, present characteristics for which data were extracted (e.g., study size, PICOS, follow-up period) and provide the citations.	Table 4
Risk of bias within studies	19	Present data on risk of bias of each study and, if available, any outcome level assessment (see item 12).	10
Results of individual studies	20	For all outcomes considered (benefits or harms), present, for each study: (a) simple summary data for each intervention group (b) effect estimates and confidence intervals, ideally with a forest plot.	10
Synthesis of results	21	Present results of each meta-analysis done, including confidence intervals and measures of consistency.	NA
Risk of bias across studies	22	Present results of any assessment of risk of bias across studies (see Item 15).	11
Additional analysis	23	Give results of additional analyses, if done (e.g., sensitivity or subgroup analyses, meta-regression [see Item 16]).	NA
**DISCUSSION**			
Summary of evidence	24	Summarize the main findings including the strength of evidence for each main outcome; consider their relevance to key groups (e.g., healthcare providers, users, and policy makers).	10-12
Limitations	25	Discuss limitations at study and outcome level (e.g., risk of bias), and at review-level (e.g., incomplete retrieval of identified research, reporting bias).	10-12
Conclusions	26	Provide a general interpretation of the results in the context of other evidence, and implications for future research.	12-13
**FUNDING**			
Funding	27	Describe sources of funding for the systematic review and other support (e.g., supply of data); role of funders for the systematic review.	13

From: Moher D, Liberati A, Tetzlaff J, Altman DG, The PRISMA Group (2009). Preferred Reporting Items for Systematic Reviews and Meta-Analyses: The PRISMA Statement. PLoS Med 6(6): e1000097. doi:10.1371/journal.pmed1000097

eTable 2. Critical assessment including sub-questions

	Jacklin PB, 2017, UK	*Danyliv A, 2016, Ireland*	*Chen PY et al., 2015, Singapore*	*Coop C, 2015, New Zealand*	*Marseille E, 2013, India and Isreal*	*Werner EF, 2012, US*	*Mission JF, 2012, US*	*Lohse N, 2011, India and Israel*	*Round JA, 2011, UK*	*Nicholson WK, 2005, US*	*Poncet B, 2002, France*
1 Was a well-defined question posed in answerable form?	100%	100%	100%	100%	75%	100%	100%	75%	100%	100%	75%
1.1 Did the study examine both costs and effects of the service(s) or programme(s) over an appropriate time horizon?	yes	yes	yes	yes	yes	yes	yes	yes	yes	yes	yes
1.2 Did the study involve a comparison of alternatives?	yes	yes	yes	yes	yes	yes	yes	yes	yes	yes	yes
1.3 Was a perspective for the analysis	yes (Healthcare)	yes (Healthcare)	yes (payers')	yes (Healthcare)	no (it seemed to be from a	yes(healthcare)	yes(societal)	no	yes(population)	yes(societal)	no

stated and was the study placed in any particular decision-making context?					healthcare perspective)						
1.4 Were the patient population and any relevant subgroups adequately defined?	yes	yes	yes	yes	yes	yes	yes	yes	yes	yes	yes
2 Was a comprehensive description of the competing alternatives given? (i.e. can you tell who did what to whom, where, and how often?)	100%	100%	100%	100%	100%	100%	100%	100%	100%	100%	100%
2.1 Were any relevant	no	no	no	no	no	no	no	no	no	no	no

alternatives omitted?											
2.2 Was (should) a 'do nothing' alternative (be) considered?	yes	yes	yes	no	yes	yes	no	yes	yes	yes	yes
2.3 Were relevant alternatives identified for the patient subgroups?	yes	N.A.	N.A.	N.A.	yes	N.A.	N.A.	yes	N.A.	N.A.	N.A.
3 Was the effectiveness of the programmes or services established?	100%	83%	75%	75%	75%	100%	25%	75%	83%	50%	100%
3.1 Was this done through a randomized controlled clinical trial? If so, did the trial protocol reflect what would happen in	N.A.	yes	N.A.	N.A.	N.A.	N.A.	N.A.	N.A.	yes	N.A.	N.A.

regular practice?											
3.2 Were effectiveness data collected and summarized through a systematic overview of clinical studies? If so, were the search strategy and rules for inclusion or exclusion outlined?	yes	Cannot tell	yes; no	yes; no (directly from two systematic reviews)	cannot tell	yes; yes	cannot tell	yes; no	yes; no (meta-analysis)	no	yes; yes
3.3 Were observational data or assumptions used to establish effectiveness? If so, were any potential biases recognized?	yes; yes	yes; yes	yes; yes	yes; yes	yes; yes	yes; yes	no	yes; yes	yes; yes	yes; yes	yes; yes

4 Were all the important and relevant costs and consequences for each alternative identified?	83%	83%	50%	33%	50%	33%	33%	67%	17%	83%	50%
4.1 Was the range wide enough for the research question at hand?	yes	yes	yes	yes	yes	yes	yes	yes	cannot tell	yes	yes
4.2 Did it cover all relevant perspectives? (Possible perspectives include those of patients and third-party payers; other perspectives may also be relevant	yes	yes	no (from a view of payers, not include the productivity losses; however, program management costs were included)	no (cost of health sector not included)	cannot tell (perspective of the paper unclear)	no (from a healthcare perspective, did not include costs of health sector)	no (did not include costs of health sector or productivity losses)	cannot tell (perspective of the paper unclear)	no (did not include the productivity losses)	yes （maternal infant costs, physician hospital costs, loss of productivity also included)	cannot tell (perspective of the paper unclear)

depending on the particular analysis.)											
4.3 Were capital costs, as well as operating costs, included?	no; yes	no; yes	N.A. (from a payer perspective)	no	no	no	no	yes	no	cannot tell	no
5 Were costs and consequences measured accurately in appropriate physical units prior to valuation (e.g. hours of nursing time, number of physician visits, lost work-days, gained life-years)?	100%	100%	100%	100%	100%	100%	100%	100%	100%	100%	100%
5.1 Were the sources of	yes	yes	yes	yes	yes	yes	yes	yes	yes	yes	yes

resource utilization described and justified?											
5.2 Were any of the identified items omitted from measurement? If so, does this mean that they carried no weight in the subsequent analysis?	no	no	yes; yes	no	no	yes (indirect costs); yes	yes; yes (antepartum admissions for GDM-related conditions)	no	no	no	no
5.3 Were there any special circumstances (e.g. joint use of resources) that made measurement difficult? Were these circumstances	no	yes; yes	no	no	no	no	no	no	no	no	yes; yes

handled appropriately?											
6 Were costs and consequences valued credibly?	100%	100%	100%	100%	100%	100%	100%	75%	100%	75%	100%
6.1 Were the sources of all values clearly identified? (Possible sources include market values, patient or client preferences and views, policymakers' views, and health professionals' judgements.)	yes	yes	yes	yes	yes	yes	yes	no	yes (third-party payer perspective)	no	yes
6.2 Were market values employed for changes	yes	yes	yes	yes	yes	yes	yes(CPI)	yes	yes	yes	yes

involving resources gained or depleted?											
6.3 Where market values were absent (e.g. volunteer labour), or market values did not reflect actual values (e.g. clinic space donated at a reduced rate), were adjustments made to approximate market values?	N.A.; yes; yes	N.A.; yes; yes	N.A.; yes; yes	N.A.; yes; yes	N.A.; yes; yes	N.A.; yes; yes	N.A.; yes; yes	N.A.; yes; yes	N.A.; yes; yes	N.A.; yes; yes	N.A.; yes; yes
6.4 Was the valuation of consequences appropriate for the question posed (i.e. has	yes	yes	yes	yes	yes	yes	yes	yes	yes	yes	yes

the appropriate type or types of analysis—cost-effectiveness, cost–benefit—been selected)?											
7 Were costs and consequences adjusted for differential timing?	100%	100%	50%	N.A.	50%	100%	50%	50%	100%	50%	N.A.
7.1 Were costs and consequences that occur in the future 'discounted' to their present values?	N.A. (no need to discount)	yes	yes	N.A. (no need to discount)	yes	yes	yes	yes	yes	yes	N.A. (no need to discount)
7.2 Was any justification given for the discount rate(s) used?	yes	yes	no	N.A. (no need to discount)	no	yes	no	no	yes	no	N.A.(no need to discount)

8 Was an incremental analysis of costs and consequences of alternatives performed?	100%	100%	100%	0%	100%	100%	100%	100%	100%	100%	100%
8.1 Were the additional (incremental) costs generated by one alternative over another compared to the additional effects, benefits, or utilities generated?	yes	yes	yes	no	yes	yes	yes	yes	yes	yes	yes
9 Was uncertainty in the estimates of costs and consequences adequately characterized?	100%	100%	83%	50%	83%	100%	83%	25%	100%	0%	17%

9.1 If patient-level data on costs or consequences were available, were appropriate statistical analyses performed?	yes	yes	cannot tell whether patient-level data were available or not, supplementary table unavailable or non-existent)	N.A. (no patient-level data)	N.A. (no patient-level data)	N.A. (no patient-level data)	N.A. (no patient-level data)	no	yes	No (no sensitivity analysis was conducted)	N.A. (no patient-level data)
9.2 If a sensitivity analysis was employed, was justification provided for the form(s) of sensitivity analysis employed and the ranges or distributions of values (for key study parameters)?	yes; yes	yes; yes	yes; yes	no; no (only GDM prevalence and OGTT sensitivity and specificity analyzed; the Sen and Spec changed simultaneously)	yes; no	yes; yes	yes; no	no	yes; yes	N.A.	no; yes

9.3 Were the conclusions of the study sensitive to the uncertainty in the results, as quantified by the statistical and/or sensitivity analysis?	yes	yes	yes	yes	yes	yes	yes	no	yes	N.A.	no
9.4 Was heterogeneity in the patient population recognized, for example by presenting study results for relevant subgroups?	yes	N.A.	N.A.	N.A.	Yes (people in India and Israel)	N.A.	N.A.	Yes (people in India and Israel)	Yes (low-high risk population)	N.A.	no
10 Did the presentation and discussion of study results include all	100%	100%	100%	100%	83%	75%	67%	33%	83%	17%	83%

issues of concern to users?											
10.1 Were the conclusions of the analysis based on some overall index or ratio of costs to consequences (e.g. cost-effectiveness ratio)? If so, was the index interpreted intelligently or in a mechanistic fashion?	yes	yes	yes	yes	yes	yes	yes	yes	yes	yes	yes
10.2 Were the results compared with those of others who have investigated the same question? If so, were	yes	yes	yes	yes	no	cannot tell (claimed to be the first comprehensive decision analysis)	no	no	yes	no	yes

allowances made for potential differences in study methodology?											
10.3 Did the study discuss the generalizability of the results to other settings and patient/client groups?	yes	yes	yes	yes	yes	no	no	no	no	no	no
10.4 Did the study allude to, or take account of, other important factors in the choice or decision under consideration (e.g. distribution of costs and	yes (with or without high risks)	yes (screen uptake rate)	yes (prevalence of GDM)	yes (some regions would add a simple blood test to avoid stigmatization)	yes (incidence of GDM and post-partum intervention)	yes (early and intensive intervention)	yes (cost)	yes (GDM prevalence)	yes (test acceptance)	no	yes (cost)

consequences, or relevant ethical issues)?											
10.5 Did the study discuss issues of implementation, such as the feasibility of adopting the 'preferred' programme given existing financial or other constraints, and whether any freed resources could be redeployed to other worthwhile programmes?	yes	yes	yes	yes	yes	yes	yes	no	yes	no	yes
10.6 Were the implications of	yes	yes	yes	yes	yes	yes	yes	no	yes	no	yes

uncertainty for decision-making, including the need for future research, explored?	

eTable 3. Treatment/intervention effects used in the baseline analyses of each study

	Jacklin PB, 2017, UK	***Danyliv A, 2016, Ireland***	***Chen PY et al., 2016, Singapore***	***Coop C, 2015, New Zealand***	***Marseille E, 2013, India and Israel***	***Werner EF, 2012, US***	***Mission JF, 2012, US***	***Lohse N, 2011, India and Israel***	***Round JA, 2011, UK***	***Nicholson WK, 2005, US***	***Poncet B, 2002, France***
(present form)	Relative risk (RR)				relative risk reduction (RRR)	relative risk reduction:	treat/no treat		treat/no treat		
PIH/PET in pregnancy		0.63079 (Log-normal)									
Prematurity		0.80990 (Log-normal)			0.422						
NICU admissions	0.77	0.7739 for term babies (Log-normal); no effect for preterm babies				1 (0.5-1.06)					
Shoulder dystocia	0.41	0.37095 in total; 0.33637 after vaginal delivery (Log-normal)			0.6	0.4 (0.21-0.75)	0.105/0.03885 if macrosomia; 0.016/0.00592 if no macrosomia				

T2DB reduction: maternal			0.4			
T2DB reduction: Child			0.4			
Fetal hyperinsulinemia			0.224, 0.2 for India and Israel			
Preeclampsia	0.46		0.455	0.65 (0.44-0.88)	0.136/0.086 in G1; 0.0679/0.0312 in G2	0.03/0.05
Cesarean	0.88	0.79283 (Log-normal)	0.14	1 (0.72-1.02)	0.338/0.338 in G1; 0.228/0.180 in G2	0.29/0.33
Preterm birth				1 (0.53-1.23)		
Macrosomia (child)			0.57		0.16/0.0656 in G1; 0.15/0.069 in G2	
Induction of labour	1.16		0.14 for Israel			0.33/0.28
Jaundice (child)	0.83		0.256			0.09/0.09
Admission to neonatal nursery			0.27			0.71/0.66

Hypoglycemia		0.2	
Permanent brachial plexus injury (PBPI)		0.905	
Hydramnios		0.353	
Breech presentation		0.356	
Gestational hypertension		0.356	
Complication risk reduction from glucose management	40 (20-60)		
Serious perinatal complications			0.02/0.05

Table S4. Baseline utilities in each study

	Jacklin PB, 2017, UK	*Danyliv A, 2016, Ireland*	*Chen PY et al., 2016, Singapore*	*Coop C, 2015, New Zealand*	*Marseille E, 2013, India and Isreal*	*Werner EF, 2012, US*	*Mission JF, 2012, US*	*Lohse N, 2011, India and Israel*	*Round JA, 2011, UK*	*Nicholson WK, 2005, US*	*Poncet B, 2002, France*
Utilities (QALY/year)	lifetime QALY decrement										
Maternal death	25 (weight 0.08)										
Stillbirth: maternal		0.92	0.92 (10				0.92				
Stillbirth: child		0	0			0	0		25 discounted loss in total	0	
Maternal pregnancy									treat/no treat: 0.72/0.70		
Maternal 3 months postpartum									treat/no treat: 0.79/0.78		
After prematurity: maternal		0.9548 (first year)									

After prematurity: child		0.96		0.96			
After PBPI (permanent brachial plexus injury): child		0.6 (Beta)		0.87	0.6; 0.99 for transient BPI	11 loss in total; 0.0017 if resolves within 2 months	(moderated morbidity) 0.7
Preeclampsia			0.99				
LSCS, lower segment cesarean section			selective: 0.99; Emergency: 0.95 (10 years)		0.99 for mother		
Macrosomia			0.8 (after 55 age)				
Shoulder dystocia and transient brachial plexus injury	0.20 (weight 0.73)		0.99 (4-5 months)			0.2	
Hyperbilirubinemia			0.7(1 week)		1		
Hypoglycemia			0.7(1 week)		1		
NICU admissions (neonatal intensive care unit)			0.5 (2 months)		1		
Hysterectomy							0.9
DALYs							

Perinatal adverse events	Birth trauma: 0.20 (weight 0.20)	no intervention: 0.23 for India and Israel; with intervention: 0.16 for India and 0.17 for Israel	
T2DB: maternal		11.2 for India and 14.3 for Israel	0.65
T2DB: child		13.7 for India and 16.1 for Israel	with iGDM progress to diabetes in 15 years: 0.257 (0.05-0.4)/0.384 (0.2-0.5)

DALY, disability of adjusted life year
iGDM, infant of gestational diabetic mother
QALY, quality adjusted life year
T2DB, type 2 diabetes mellitus
